# Machine-learning based routing of callers in an Israeli mental health hotline

**DOI:** 10.1186/s13584-022-00534-9

**Published:** 2022-06-03

**Authors:** Akiva Kleinerman, Ariel Rosenfeld, Hanan Rosemarin

**Affiliations:** Bar-Ilan University, Ramat-Gan, Israel

**Keywords:** Call routing, Contact centers, Mental health, Online scheduling, Monte Carlo tree search

## Abstract

**Background:**

Mental health contact centers (also known as Hotlines) offer crisis intervention and counselling by phone calls and online chats. These mental health helplines have shown great success in improving the mental state of the callers, and are increasingly becoming popular in Israel and worldwide. Unfortunately, our knowledge about how to conduct successful routing of callers to counselling agents has been limited due to lack of large-scale data with labeled outcomes of the interactions. To date, many of these contact centers are overwhelmed by chat requests and operate in a simple first-come-first-serve (FCFS) scheduling policy which, combined, may lead to many callers receiving suboptimal counselling or abandoning the service before being treated. In this work our goal is to improve the efficiency of mental health contact centers by using a novel machine-learning based routing policy.

**Methods:**

We present a large-scale machine learning-based analysis of real-world data from the online contact center of ERAN, the Israeli Association for Emotional First Aid. The data includes over 35,000 conversations over a 2-years period. Based on this analysis, we present a novel call routing method, that integrates advanced AI-techniques including the Monte Carlo tree search algorithm. We conducted an experiment that included various realistic simulations of incoming calls to contact centers, based on data from ERAN. We divided the simulations into two common settings: standard call flow and heavy call flow. In order to establish a baseline, we compared our proposed solution to two baseline methods: (1) The FCFS method; and (2) a greedy solution based on machine learning predictions. Our comparison focuses on two metrics - the number of calls served and the average feedback of the callers (i.e., quality of the chats).

**Results:**

In the preliminary analysis, we identify indicative features that significantly contribute to the effectiveness of a conversation and demonstrate high accuracy in predicting the expected duration and the callers’ feedback. In the routing methods evaluation, we find that in heavy call flow settings, our proposed method significantly outperforms the other methods in both the quantity of served calls and average feedback. Most notably, we find that in the heavy call flow settings, our method improves the average feedback by 24% compared to FCFS and by 4% compared to the greedy solution. Regarding the standard-flow setting, we find that our proposed method significantly outperforms the FCFS method in the callers’ average feedback with a 12% improvement. However, in this setting, we did not find a significant difference between all methods in the quantity of served-calls and no significant difference was found between our proposed method and the greedy solution.

**Conclusion:**

The proposed routing policy has the potential to significantly improve the performance of mental health contact centers, especially in peak hours. Leveraging artificial intelligence techniques, such as machine learning algorithms, combined with real-world data can bring about a significant and necessary leap forward in the way mental health hotlines operate and consequently reduce the burden of mental illnesses on health systems. However, implementation and evaluation in an operational contact center is necessary in order to verify that the results replicate in practice.

## Background

### Introduction

Mental health conditions such as depression and anxiety are a major global health concern and their prevalence across the globe, and specifically in Israel, is growing rapidly [1, 2]. According to the World Health Organization (WHO), there has been a 13% rise in mental health conditions in the last decade [1]. The need for effective and timely mental health intervention is currently unmet [3]. According to the WHO, between 76 and 85% of people who need mental health support in low and middle-income countries do not receive it [1]. In America, 60% of youth suffering from depression did not receive any mental health support in 2017–2018 [4]. Recently, since the Covid-19 pandemic began to spread rapidly in March 2020, the number of people seeking help with a mental health issue or concern has grown steeply [2, 5]. Specifically in Israel, there has recently been an unprecedented increase in mental health related issues and demand for help [2, 6].

Unfortunately, while many mental health conditions can be effectively treated and mitigated through counseling, many choose to avoid in-person appointments due to stigma and difficulty in self disclosure [7]. In addition, those who do seek help often find it extremely difficult to obtain an appointment with a qualified personal due to shortages in manpower and the (sometimes very substantial) associated costs [3].

Against this background, immediate, accessible, free and anonymous mental health help is provided by various organizations which offer counseling and emotional support through telephone calls and, more recently, textual chats [8]. These organizations operate in what is often called “hotlines” or “contact centers”, which have shown to attract more callers over time [7], and proved to be effective in providing mental health first-aid (e.g., [8–10]). These contact centers commonly focus on suicide prevention and other short-term counselling interventions which are not intended to replace the typically long-term patient-therapist relationship. As such, most callers to these contact centers are treated by the first available counselor and by different counselors each time they call.

Unfortunately, contact centers are fundamentally limited by the available counselling staff and are overwhelmed with the ever-increasing calls and chat requests [11]. Specifically in Israel, the need for mental health contact centers has recently grown notably, and this need peaks periodically due to the fragile security situation: After March 2020, when the COVID-19 virus has begun to spread in Israel, the number of calls to ERAN has more than tripled in comparison to the preceding period [2] and has peaked during the violent Israeli-Palestinian events in May 2021 [6].

The increase in the need for mental health support results in prolonged waiting times in the contact centers, which can lead to high abandon rates, and to short, ineffective or unsatisfying counselling sessions. As such, the central objective of these contact centers should be getting the right caller to the right counselor at the right time.

In this work, we tackle the challenge of efficiently routing callers to counselors in mental health contact centers (MHCCs for short) by utilizing artificial intelligence techniques, in order to increase the service quality and decrease the abandonment of callers.

The problem of caller abandonment and low service quality is also common in general contact centers, such as technical support call centers for cellular providers. However, in most general contact centers, the service process and its outcomes are more straightforward and well understood than that of MHCCs. Specifically, callers to a technical support call centers for a cellular provider commonly self disclose the required service prior to being directed to an agent (e.g., they want to add/remove a service, they have a financial query, etc.). As such, many characteristics of the interaction can be easily estimated and accounted for (e.g., call duration, success rate, etc.). On the other hand, callers to MHCCs commonly do not provide any significant information prior to being directed to a counselor and require a much more personalized treatment which varies significantly from one caller to the other (e.g., in terms of call time, counseling strategies, etc.).

In this work, we present a large scale machine-learning based analysis of real-world data from the MHCC of ERAN^1^, the Israeli association for emotional first aid. We demonstrate how the data can be utilized in order to predict the quality and the duration of the chats. Our analysis provides *actionable* insights that can be useful for MHCCs in Israel and worldwide. We then present an AI-based solution for call routing, that leverages our prediction models and a Monte Carlo tree search (MCTS) algorithm, an advanced AI search algorithm [12]. We evaluate our proposed solution and compare it to common and state-of-the-are methods for routing calls in contact centers. For the evaluation, we created realistic simulations of calls arrival during a shift of a MHCC based on data from ERAN. We divided the simulations into normal call flow and heavy call flow simulation and evaluated our proposed solution in each setting with 1000 simulations. We show that our solution can bring about a significant increase in the counselling quality as well as increase the number of calls being served during a shift. Finally, we discuss our results and the possible implications and limitation of the results. To the best of our knowledge, this is the first work that addresses the problem of call routing in the mental health domain.

### Related work

Contact centers are an increasingly important part of costumer-service in several organizations. They employ millions of agents around the world and serve billions of customers in a wide range of industries, including private companies, government agencies and emergency services [13–15]. Initially, the primary communication channel in many contact centers was through telephone calls, and therefore they were often named “call centers”. However, in the past two decades, many organizations have started offering service through online chats, staffed by human or automated agents. In this work, we adopt the more general term “contact centers” or “hotlines” interchangeably, which includes both types of communication channels [16]. For simplicity, we will refer to any type of inbound communication as a “call” and the person who initiates this call as “caller”.

A contact center applies some type of method for routing the calls to the available agents or to a waiting queue. The selected routing method can have a substantial effect on contact center’s objectives. Specifically, previous research has shown that the routing method has the potential of reducing the caller’s abandon rate (meaning the ratio of abandoned calls from all received calls), the average waiting time to an answer and the service-time [15–18]. However, the routing itself is highly complex for three main reasons: First, different calls may be associated with different expected service time and may differ in their “importance” to the organization; Second, during the time-point of routing the call, the system’s information is incomplete. Specifically, the system does not know the arrival times and the types of future incoming calls, and therefore the a short-term optimal routing decisions may turn out to be sub-optimal in the long run.

While we are unaware of any work that investigated routing methods for MHCCs, prior work in the field has laid out foundations which can be used to that end. Specifically, Althoff et. al. [19] have investigated post-hoc indicators for the quality of texting-based conversations to MHCCs. They applied AI-techniques to analyze chats, and discovered conversation strategies that are likely to improve the conversation outcomes. Closely related to our work is the research of Grigorash et al. [20]. In their work, the authors apply clustering and supervised machine learning algorithms in order to identify types of callers in MHCC. Specifically, they use the popular k-mean clustering algorithm [21] in order to cluster the callers according to the following features: (1) number of calls; (2) mean call duration, and (3) standard deviation of call duration. They define five type of callers according to the identified clusters. However, they do not propose a method for implementing routing of callers to agents. In addition, the identification of caller type is based on previous 8 and 16 initial calls of a caller, data which is unfortunately unavailable in many cases, as is the case in ERAN.

Our work builds on these advances and applies machine learning for designing a solution for routing calls in MHCCs. Unlike previous work, we design prediction models that predict characteristics of a chat *before and during* the chat, for a given pair of caller and agent, thus promoting a real-time, adaptive and fully personalized routing approach.

### Problem definition

We start by defining the main components of a MHCC.

A contact center is a system that aims to provide some type of service to *callers*. A contact center continuously receives incoming calls and applies a *routing method* in order to direct the calls to a staff of *agents*, who provide the requested service.

The callers: a call $c_i$ is associated with a list of features describing the call and the caller such as the call arrival time, the caller’s age and if she is a new caller or revisiting. In addition, each call is associated with the maximal time the caller is willing to wait for an answer before abandoning, which is commonly termed “patience” [22]. The “patience” of callers is generally unknown to the contact center.

The agents: the MHCC schedules in advance a group of agents in shifts. The number of agents in a shift can vary possibly according to the expected traffic of call arrivals. Each agent $a_j$ is described by a list of features describing that agent such as agent’s age and gender.

A routing method is some type of policy that matches between in incoming call and an agent. This policy generally aims to achieve some objectives of the contact center.

In this work, we assume an agent handles only a single call at any given moment, since the guidelines of both the MHCC we collaborated with (ERAN), and many other MHCCs, is to only allow a single call at a time, in order to increase the service quality.

The Objective. We conducted an interview with the manager of the internet-based branch of ERAN, a domain expert in MHCCs, who defined the two prime objectives which are shared across all MHCCs:

1. *Quantity.* The fraction of calls that were answered and treated by agents out of the calls that arrived during the shift. We denote this measure as *Quantity*. Note that this measure is the complement of the abandonment rate, which is widely used to measure contact centers performance in general.
2. *Quality*. Every individual call $c_i$ is associated with a service-quality measure, denoted $q(c_i)$. The quality of the conversations in a shift is the average of $q(c_i)$ over all calls $c_i$ in that shift. $q(c_i)$ can be estimated by various indicators, such as the callers’ explicit feedback, if exists. We denote the average quality measure as *Quality* .

According to our domain expert, in ERAN, as in most MHCCs, both of the objectives are considered equal in importance. On the one hand, the MHCC aspires to increase the quantity of treated calls, but on the other hand, the MHCC must make sure that the quality of the conversation is high. These objectives are partially conflicting, since longer chats are likely to increase the quality of the chats, but decreases the number of treated calls. We balance the objectives by simply giving them equal weights:

1 $$\begin{aligned} Obj(scenario)= \frac{1}{2} \times Quantity(scenario) + \frac{1}{2} \times Quality(scenario) \end{aligned}$$

where both the Quantity and Quality are normalized and scaled on the range of 0 to 1.

The problem of matching agents to calls in MHCCs can be therefore defined as follows: A routing system in an MHCC can, at any time during a shift, decide to match a waiting or an arriving call $c_i$, to an available agent $a_j$ for counselling. All unmatched calls are placed in the waiting queue. The MHCC aims to maximize some objective (to be defined shortly), while satisfying the following constraints:

1. Every call is handled by a single agent, and the call cannot be interrupted (it cannot be divided into two parts).
2. Each agent can only handle a single call at any given moment.
3. The agents in the shift are predefined and are not subject to change.
4. The outcomes of a call, including the duration (service time) and the caller’s feedback, are unknown in advance.
5. Callers can abandon the service while waiting. The patience of the callers varies significantly and is unknown in advance.
6. The arrival times of callers and the number of callers during the shift are unknown in advance. However, the call arrivals are assumed to follow some distribution.

## Methods

### Individualized prediction routing

In order to tackle the challenge of efficiently routing requests in MHCCs, we propose a two-phased approach we name Individualized Prediction Routing:

1. Applying Machine Learning and Natural Language Processing techniques in order to predict attributes of any potential caller-agent matching.
2. Leveraging the prediction models together with advanced routing rules to approximate the optimal routing for each caller.

**Fig. 1:**
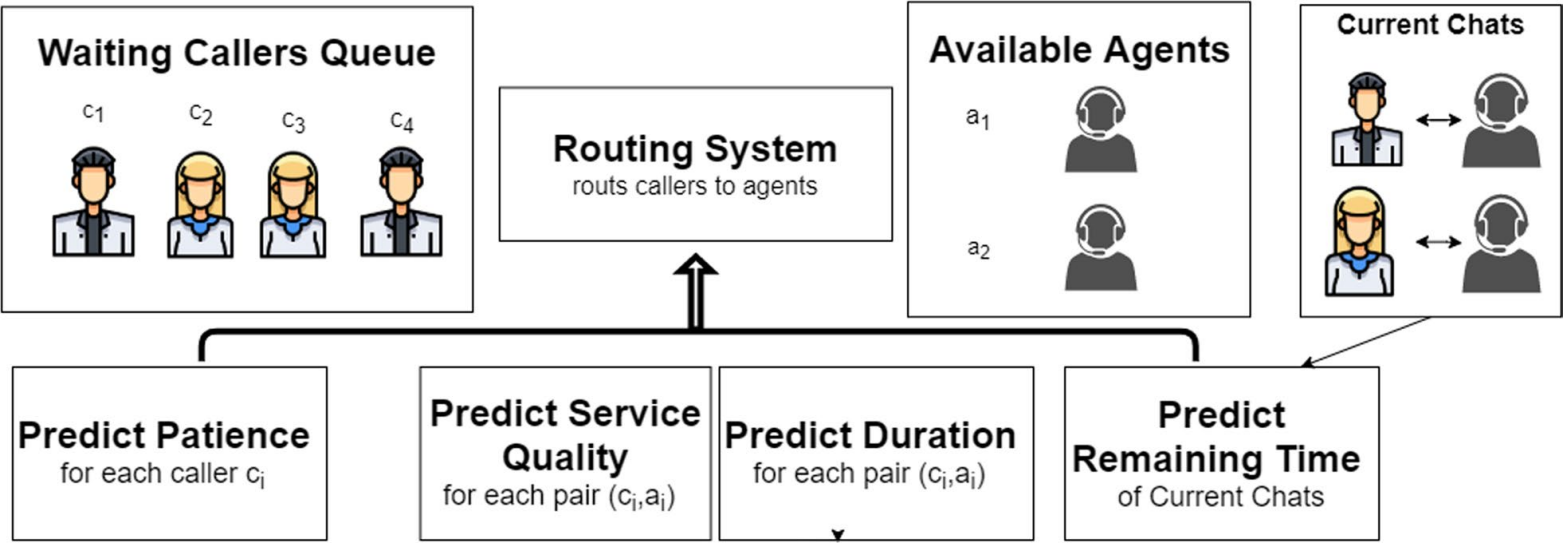
Visualisation of our proposed routing approach. The visualization illustrates the mechanism of our approach at a given moment in an MHCC. The people on the top left of the figure ($c_1, c_2, c_3, c_4$) are callers that were not answered yet. The “available agents” box presents agents that are not currently engaged in a conversation. The “current chats” box presents ongoing conversations between agents and callers who have been answered. The prediction models appear at the bottom of the figure. The three models in the left use the data from the waiting callers and the available agents. The last model, predicting remaining time, uses the data from the current conversations: describing the agents and the callers and the current chat

Figure 1 provides a high-level visualization of our approach. In this figure, the “routing system” is the main component, which directs a caller to an MHCC agent. The other rectangles represent prediction models that provide estimations to the “Callers’ triage” component regarding the chat’s estimated outcomes and the callers patience. Below, in Sect. 2.1.1, we will describe the models and discuss their necessity. Then, we will present the routing algorithm.

#### Prediction models

Our approach utilizes four prediction models:

1. Prediction of chat *duration* prior to chat beginning, meaning that the prediction model only uses the data existing prior to the chat.
2. Prediction of chat’s *quality* prior to chat beginning.
3. Prediction of the caller’s *patience*, meaning the time the caller is willing to wait to be served before abandoning.
4. Prediction of the *remaining time* of a chat: this model predicts the remaining time of chat, from an ongoing chat. In contrast to the first prediction model, this model predicts the duration of the chat *after* the chat has begun. Therefore, the model can utilize data describing the beginning of the chat.

The importance of the first two prediction models for the routing decision system is clear: the routing of the call is only performed once and before the chat begins, therefore the system must predict the outcomes (duration and feedback) of the possible assignment of caller to agent with the available information prior the chat. The third model is also clearly essential since it is very common that more than one caller is waiting to be served and the system is likely to benefit from prioritizing impatient callers. The fourth model predicts the future availability of the agents. Predicting the *remaining time* of a chat, after the chat has begun, can potentially improve the estimation of the end time of that chat. Specifically, the text of the chat up to a certain time-point may includes important features, such as specific words used by the agent or caller and the structure of the conversation, and these features can be useful predictors for the remaining time of the conversation, as we will see in our case study (Sect. 2.2) To the best of our knowledge, this is the first work in the field of contact centers (not just MHCCs) that suggests predicting the *remaining time* of calls for routing calls.

In the following subsection, we provide the routing algorithm used in our approach.

#### Routing algorithm

We devise the following Routing Algorithm:

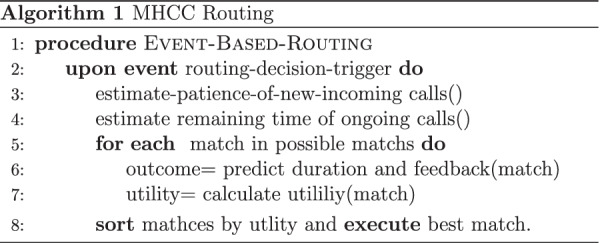



The routing algorithm is event-based, meaning that the routing method is triggered by an event- as defined in row 2. The “routing-decision-trigger” can be defined by the MHCCs needs and abilities. It makes sense that an arrival of new caller or an occupied agent who became available to receive a call will trigger the process. The *estimate patience of new incoming calls()* function, called in row 3, uses the fourth prediction model mentioned above for estimating the callers patience. Similarly, the *estimate remaining time of ongoing calls()* function, referenced in row 4, uses the third prediction model for estimating the remaining time of the chats. The *predict duration and feedback()* function, called in row 6, evaluates the duration and the prediction of the model with the first two prediction models discussed in Sect. 2.1.1. In row 5, the algorithm iterates over the possible routing actions. The possible matches include every possible match between available agents and waiting callers, and also, possibly, a null action, meaning that the system decides not to route a call. In row 7, the algorithm applies a method for evaluating the utility of a match, given these various predictions. Finally, the algorithm pairs the match with the highest utility.

### Case study: ERAN

In this study, we use the online chats dataset of the Israeli Association for Emotional First Aid, ERAN. ERAN is a volunteer based organization that provides initial response and emotional support on the phone and online. The organization was established in 1971, and today it includes over 1450 volunteers. In the past year, ERAN provided assistance to more than 365,000 calls, including 1,120 suicidal inquiries [23]. The conversations in ERAN are mostly in Hebrew, but ERAN also provides support in additional languages such as Arabic and English.

#### The data

In this work, we use a dataset of online chats, since the online chat’s are well-documented in comparison with the telephone call dataset. Specifically, the online chat dataset includes explicit feedback from the callers and a full and detailed transcript of each conversation which can significantly improve the prediction of various outcomes of the calls, as we present below (Sect. 2.2.4). Nevertheless, we continue using the terminology defined above, meaning that the visitor of the chat service is termed “caller”.

The dataset includes about 35,000 records of online chats that took place between August 2019 and July 2021. Each online chat record includes several attributes. These attributes can be divided into three main groups:

1. *Chat meta-data*, or basic information, such as date and time of a chat, the duration of the chat and the callers’ waiting time.
2. A full transcript of the chat, including the text of each message, and the exact time that the message was sent.
3. A unique ID of the agent and the caller, and a few attributes of callers and agents: minimal information is collected about the *caller* prior to chat, as described below (Sect. 2.2.2) and a few attributes describing the caller’s access point and her device type (Mobile/Desktop) are included.

In addition, the dataset also includes a brief description about each *agent* including the following attributes: the agent’s age and gender, years of experience in ERAN and whether the agent has an additional role in the organization.

#### The online chat process in ERAN

**Fig. 2:**
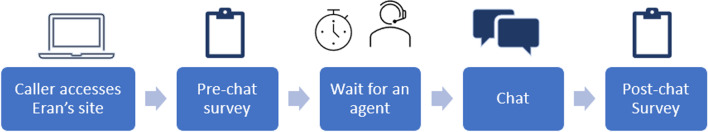
The basic process of chat with ERAN’s service. The figure shows the five main stages of a call using ERAN’s online service. The post-chat survey is optional

The online chat service is accessed through ERAN’s official website: *https://www.eran.org.il/*. The service is open 18 hours a day (8:00AM to 2:00AM). Before the chat begins, the caller fills a short pre-chat survey. In order to maintain the anonymity of the callers, which is a fundamental aspect in mental health services, ERAN requests only minimal information about the caller: (1) A username (nickname); (2) The callers age range; (3) The callers’ needs and expectation from the conversation (categorical); (4) An optional brief description of the problem and the background that led the caller to contact ERAN . In practice, most callers (58%) do not provide such a background. After filling the pre-chat survey, the caller is placed in the waiting queue and the system periodically informs them on their current position in the list. Callers commonly abandon the service before being answered, and specifically when the queue is relatively highly loaded. The agents, in turn, pull chat requests one-by-one, in the order arrival, practically implementing the FCFS heuristic. The caller is then informed that an agent is available and the conversation begins. After the chat ends, the caller is given an option of filling a post-chat survey with a 5 point Likert scale , debriefing them about their general experience and specific aspects of the chat. [24]. Only about a quarter of the caller provide such a feedback. Figure 2 illustrates this process.

#### Predicting the callers’ feedback

As mentioned above, callers can provide feedback regarding their satisfaction from the chat. In this work, we use this feedback to measure the quality of the call, since it is the only measure that accurately reflects the callers’ feeling and mental state at the end of conversation, which is the primary concern of the hotline. Recall, the feedback is provided on a 5 point Likert scale.

Prior to building the prediction model, we first analyzed the interaction of the feedback inputs with other features. Interestingly, we found that the age of the agent is negatively correlated with the feedback, meaning that older agents receive worse feedbacks in general. Specifically, we found that while the average feedback for all agents is 4.19, the average feedback for agents over the age of 65 is 3.96. In order to examine if the feedback is effected by the age difference between the agent and caller, we divided the *callers* by age groups and analysed the average feedback of older agents (above age 65) with different caller age groups. We found that across all caller age groups, the callers gave worse feedback to older agents on average. However, older callers (over age 65) were more satisfied with older agents than any other caller age group (mean= 4.15). This indicates that although callers generally prefer younger agents, older callers are more accepting of older agents. A possible explanation for this fact is that older agents are often type slower than others, and therefore the chat interaction may be unsatisfying.

We then developed a machine learning classification based prediction model for predicting the explicit feedback of callers who filled the feedback survey. As our final goal is to effectively route the caller, we only used attributes available *before* the chat begins. In order to benchmark the performance of the model, we use a relatively naïve baseline prediction: that baseline method predicts the feedback of an agent’s chat as the average feedback of all previous chats.

Features: For each chat that received feedback, we collected several features. We first obtained the basic features describing the caller and the agent as described above in Sect. 2.2.1. Then, we processed the previous chats the agent and the caller (if she has visited before) in order to produce various features describing their *chat style*. These features included the average delay of response , the average ratio between the number of words of the agent and the caller in previous chats, and the average ratio of sentences. The features aim to reflect that counselling skills of the counsellor that include active listening. These skills are associated with positive outcomes of the conversation. In addition to the chat style features, we collected the agent’s average received feedback and the caller’s average provided feedback.

In order to improve the results of our prediction models, we used a few different feature selection methods. We found that backward feature elimination, that iteratively finds the features with the highest significance level (*P*-value) [25], brought the best results and therefore we used this feature selection method in all models, including the feedback prediction. At the end of the process, we selected 14 features out of 28. In Table 1 we show the most influential features, ordered by the Pearson correlation value between the feature and the feedback.

**Table 1:** Prominent features used in the feedback prediction model. The features are ordered by their correlation with the feedback score. Interestingly, the age of the agent is negatively correlated with the the feedback, meaning that younger agents receive better feedback. Unsurprisingly, the average feedback of the agent in previous calls is correlated with the feedback at the current call. In addition, the average ratio between agent messages and caller messages in previous calls is correlated with feedback, meaning that agents who generally write many messages are likely to receive a better feedback

Feature	Correlation
Agent age	− 0.18
Agent average feedback	0.176
Agent average message ratio	0.16
Agent’s number of chats during the shift (before current chat)	− 0.145

Next, we tested the performance of a variety of machine learning algorithms to identify the best performing model in terms accuracy. We focused on the balanced-accuracy metric [26], since the labels were highly unbalanced. We found that the random-forest algorithm performed best in this prediction task (balanced accuracy= 0.758, F1-score= 0.743). In Table 2 we present the performance of various machine learning algorithms, sorted by their accuracy. We used the 10-cross-fold validation for measuring the performance. We repeated the process 100 times and averaged the results.

**Table 2:** Performance of various machine learning prediction models for prediction of the feedback. The random forest significantly outperformed all other prediction methods

Metric		
algorithm	Balanced accuracy	F1-score
Random forest	0.758	0.743
Decision tree	0.545	0.547
Ada-Boost Classifier	0.368	0.366
Baseline (agent’s average)	0.281	0.302
Logistic Regression	0.250	0.228

#### Prediction of chat duration

As mentioned before, our approach integrates two types of duration estimation model: (1) a model for predicting duration prior to chat in order to roughly estimate the chat’s total duration; and (2) a remaining time predictor in order to estimate when the occupied agent will become available. In the following subsections, we will describe each model separately.

#### Predicting duration prior to the chat

Similar to process described above (Sect. 2.2.3), we used backward feature elimination for feature selection, which selected 28 features from 35 initial features. Table 3 lists the most prominent features and their correlation with the chats’ duration. Unsurprisingly, the two most prominent features were the average duration of previous chats of the agent and that of the caller.

**Table 3:** Prominent features used in the duration prediction model, ordered by their correlation with the duration of the chat. Unsurprisingly, the average duration of the agent and caller in previous calls is correlated with the duration of the current call

Feature	Correlation
Agent average chat duration	0.178
Caller average chat duration	0.136
Agent’s number of chats in current shift	0.10
Agent average message length	0.09

After that, we tested various machine learning regression algorithms. In order to benchmark the performance of the model, we use a baseline method that predicts the duration of a chat for a given agent as the average duration the previous chats. We found that the baseline approach scored 455.198 in the MSE measure while a linear regression model [27] scored 399.88 (14% improvement). The linear regression model also outperformed other common regression models (AdaBoostRegressor [28] =428.62 , ARDRegressor [29] = 435.96 ).

#### Predicting a chat’s remaining time

For predicting the remaining time of a chat we integrate two prediction models: the first is a regression prediction model that predicts the time remaining for a chat from a given point in the middle of a chat, and a binary classifier that predicts if the chat is expected to end shortly, in *k* minutes. see Appendix A for more details about the integration of both models and for the justification of the necessity of both models.

The features for these models include lexical features describing the agent’s and caller’s messages, these features provide a conceptual representation of the chat. We used a “bag-of-words” representation of text [30] in order to describe the occurrences of words within the agent’s messages and the caller’s messages, meaning that each possible word is a feature and the number of occurrences of the word is that feature’s value. We pre-processed the text by first braking it down into individual terms based on white spaces (known as *tokenization*). Later, we used Yap [31], a morphological and syntactic analysis tool for Hebrew texts, in order to couple similar words and analyze the text. Prior to the application of the feature selection process described above (Sect. 2.2.3), we applied various n-gram models and other lexical analysis in order to produce valuable features. We then reduced the number of features by eliminating rare words and phrases. We found that specific words and sentence structures were effective indicators for a long or short remaining time. For example, using future tense in recent messages of both agent and callers is negatively correlated with the remaining time (Pearson correlation= − 0.11), meaning that the more the messages contain words in future tense, the more likely that the chat is towards the end. Similarly, the agent using the phrase “the conversation” is negatively correlated with the remaining time (probably since this type of terminology is used when concluding a conversation). Unsurprisingly, when the caller wrote “thank you” in her recent sentences, the models predicted that the conversation was very close to its end. When the caller wrote the word “no” or “but”, the conversation was likely to last for more than three minutes (correlation= 0.145 and 0.08). For more details regarding these models, see Appendix A.

#### Predicting the patience of callers

The patience of a caller is the time the caller is willing to wait for being assigned to an agent before abandoning the waiting queue. Previous literature has suggested various methods for estimating the patience of different types of callers in call centers [22]. Note that the patience is inherently different than the actual waiting time until the call is answered: while the waiting time is observed explicitly in the data, the patience of callers can only be estimated [22]. Nevertheless, the actual waiting time is correlated with the patience [22].

Following previous work (e.g. [32]), we estimated the patience of the callers by an exponential distribution. We tuned the distribution parameter ($\lambda$) for each call according to the features that were most influential on the waiting time. For this purpose, we analyzed the waiting time of callers with different features. We found, for example, that the device the caller used during the connection has a significant influence on the waiting time. Specifically, callers who access the service by a desktop computer waited about 50% more in average than those who accessed by a mobile phone (402 seconds, s.d.= 743.21, vs. 266 seconds, s.d.= 523.58). For more details regarding the patience estimation, see Appendix B.

### Evaluation

In order to evaluate our approach, we created a sequence of MHCC simulations based on real-world data from ERAN.

In order to create the simulations, we first analyzed the arrival times of callers in the ERAN service. As in similar MHCC services (e.g. [20]), the number of callers varies significantly during the week, and peaks at weekends and in weekdays’ evenings. In addition, the staffing of the agents in ERAN service depends on the time of the day, ranging from 2 agents in the morning hours (from 8:00 to 14:00) to 4 in the evening (20:00 to 24:00). Accordingly, we created two sets of simulations: 1) standard call flow, simulating shifts where the call arrivals are around the average. Specifically, the average of incoming calls per hour in ERAN is about 7, and therefor the standard call flow simulations included simulation with between 5 and 9 incoming call. This rate of calls is common in about 50% of the shifts in ERAN. 2) heavy call flow, simulating shifts where the number of call arrivals are at least 10, about 40% higher than the average call flow. This rate of calls occurs in about 28% of the shifts in ERAN, meaning that it is a relatively common setting.

In both cases, we generated the arrival of patients according a non-homogeneous Poisson process (as in similar work, e.g. [22]). We set the parameters of the distribution according to the average number of calls in ERAN at heavy call flow hours and standard-flow hours. Note that we do not consider low flow scenarios, since the routing method does not have a significant effect at this setting.

Note that a chats’ outcomes, and specifically the duration and feedback of the chats, cannot be observed in the data, since a routing mechanism must consider all possible matches between agents and callers (and not only the ones that had actually happened). Therefore, in order to simulate a chat between an agent and a caller, we collected the features of both parties and fed it to the feedback and duration prediction models described above. Then, we added to the prediction an *inaccuracy error*, which we calculated as follows: for each of the prediction model we obtained all prediction errors across the test-set. We found that the prediction errors were normally distributed using the Shapiro-Wilk’s normality test [33]. Then, we defined an error normal distributiion for each model. During the simulation, for each prediction of the models, we generated an innacuracy error from the these distribtion and added it to the prediction.

### Evaluated routing methods

In order to forecast the effect of a routing decision we apply the MCTS algorithm. MCTS has proven itself capable of achieving state-of-the-art performance in modeling both dynamic behaviour and the long-term effect of an agent’s decisions in complex environments such as the games of Go and Chess [12]. We adopted this approach since the callers’ routing is performed with unknown information of future events, and for deciding one must contemplate several different possibilities. We implemented a MCTS that is tailored for our setting and added pruning techniques and heuristics in order to optimize the search. For each routing decision, we ran 300 simulations of callers arrivals generated according to arrival distribution discussed above. We will call this method MCTS.

In addition, we used a simple routing rule that utilizes our prediction models and is inspired by a similar established routing rule that has been proven to be effective in calling centers [32]. The routing rule estimates the utility of assigning call $c_i$ to agent $a_j$ as follows:

$$\begin{aligned} utility= \frac{quality(c_i,a_j)}{ duration(c_i,a_j)\times patience(c_i) } \end{aligned}$$

The estimation of *quality*, *duration* and *patience* are all produced from the prediction models discussed above. This rule is inspired and adjusted from a similar rule, named $c_i \mu _i / \theta _i$ rule [32], that has shown to be effective in improving the performance of general contact centers. We will call this method *CMT* from now on.

We further compare our approach to the standard First-Come-First-Served (FCFS) routing method, which is the method currently used by ERAN and in many other similar MHCCs^2^.

For replication purpose, the implementation of the routing methods and the simulations are all available in: *https://github.com/AkivaSinai/MHCC*.

## Results

In each setting, we ran 1000 simulations. In each simulation, we tested the performance of each of the three routing methods in terms of *Quality*, *Quantity* and the balanced objective (average of the results).

Regarding the *standard flow setting*, we found that the MCTS routing method outperformed the FCFS method in both the *Quality* with a  12% improvement (FCFS: mean=0.681, s.d.=0.089 , CMT: mean=0.753, s.d.= 0.056 , MCTS: mean=0.766, s.d.=0.06 ) and the balanced objective with a  6% difference (FCFS: mean= 0.754, s.d.= 0.061 , CMT: mean=0.794, s.d.= 0.058 , MCTS: mean= 0.833, s.d.= 0.071, $p\le 0.01$), both differences were significant ($p\le 0.01$). However, in this setting, no significant difference was found between all methods in *Quantity* (FCFS: mean=0.832, s.d.=0.034 , CMT: mean=0.836 , s.d.= 0.031 , MCTS: mean= 0.833, s.d.= 0.029 , $p\le 0.01$) and no significant difference was found between MCTS and CMT in all objectives. These relatively similar results across methods is also not surprising, since in the standard flow setting, the number of incoming calls is smaller as are the possibilities in routing decision, and consequently the routing method has smaller impact. The results of the standard flow simulations are presented in Table 4.

**Table 4:** The results of all algorithms in the standard flow setting. both MCTS and CMT outperformed FCFS significantly in quality and the weighted-objective. No other significant differences were found

Algorithm			
objective	FCFS	CMT	MCTS
Quality	0.681	0.753	0.766
Quantity	0.832	0.836	0.833
Weighted sum	0.754	0.794	0.799

**Fig. 3:**
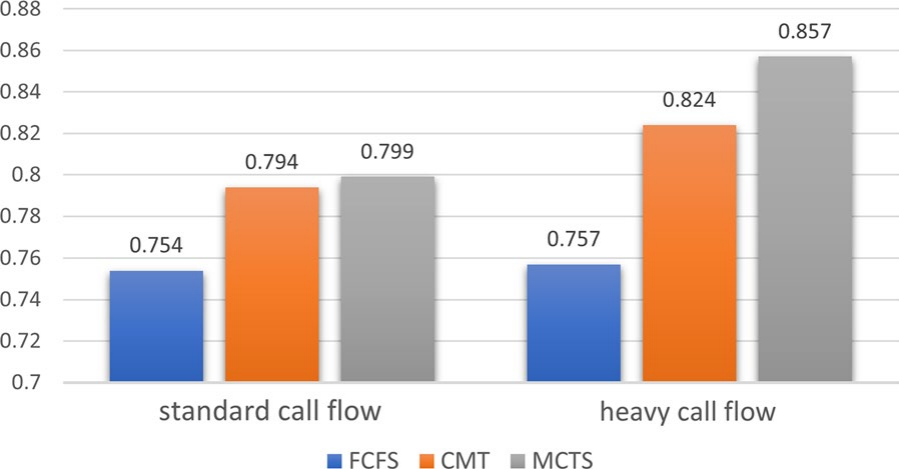
The result of the weighted-sum objective in both settings. The results show that MCTS, the proposed approach, is significantly superior to all other approaches in the heavy call flow setting. In the standard call flow setting, MCTS and CMT perform similarly and both outperform FCFS, which is the common approach in MHCCs today

In the *heavy call flow setting*, we found that the MCTS routing method significantly outperformed the other methods in all objectives, and the difference is most significant in the *Quality* measure : Specifically, regarding *Quality*, MCTS improved by 24% compared to FCFS, and by 4% compared to CMT. The difference between CMT and FCFS was also significant (FCFS: mean=0.687, s.d.=0.09 , CMT: mean=0.820, s.d.= 0.064 , MCTS: mean=0.851, s.d.=0.057 , $p\le 0.01$). Regarding *Quantity*, MCTS significantly outperformed the other methods by about 2.5% ($p\le 0.01$), but no significant difference was found between the CMT method and FCFS (FCFS: mean=0.845, s.d.=0.111 , CMT: mean=0.851, s.d.=0.102, MCTS: mean=0.873, s.d.= 0.118). Regarding the balanced objective, MCTS improved by about 13% in comparison with FCFS and by about 4% in comparison with CMT (FCFS: mean= 0.757, s.d.= 0.07, CMT: mean=0.824, s.d.= 0.051, MCTS: mean= 0.857, s.d.= 0.063, $p\le 0.01$). The FCFS result in *Quantity* is not surprising since the FCFS method assigns the longest waiting caller to a free agent, which in turn reduces the abandonment rate. The results of the heavy call flow simulation are presented in Table 5. Figure 3 presents the results of the balanced objective in both settings.

**Table 5:** The results of all algorithms in the heavy call flow setting. MCTS outperformed the other methods significantly in all objectives

Algorithm			
objective	FCFS	CMT	MCTS
Quality	0.687	0.820	0.851
Quantity	0.845	0.851	0.873
Weighted sum	0.757	0.824	0.857

### Marginal effect of each prediction model

In order to measure the influence of each of the four prediction models, we tested four additional variations of the MCTS method: In each variation we dropped one of the prediction models, and used a naive prediction in its place that simply predicts the average value. We focus our analysis on the heavy call flow setting, since this setting is more sensitive to differences in the routing method, and we only focus in the MCTS, since it is the only method that utilizes all prediction models. We ran the same simulations in all variation and measured the objectives discussed above. The results are presented in Table 6.

**Table 6:** The Marginal Results without each of the prediction models, heavy call flow setting. The first column (None) presents the result of MCTS with all components. The rest of the columns present the results of MCTS without one prediction model. We can conclude that all prediction models improved the MCTS results

Prediction dropped					
objective	None	Feedback	Duration	Patience	Remaining time
Quality	0.851	0.751	0.787	0.841	0.824
Quantity	0.873	0.802	0.846	0.800	0.822
Weighted sum	0.857	0.777	0.817	0.821	0.823

The results show that dropping each of the prediction models worsens the results, in other words, all prediction models that were used have some positive influence on the measured objectives. Specifically, the prediction of the feedback has the biggest influence on the performance of the model both the quality of the chats and the weighted objective.

### Computation time

MCTS requires many simulations that naturally increase the computation time, therefore in is not surprising that the MCTS method required significantly more computation time than the other methods. Specifically, running a single simulation of shift, required on average 13 seconds, while the other methods ran for less than a second. The running time was higher in heavy call flow hours, due to the breadth of the search tree. This means that applying MCTS for directing can increase the waiting time of the callers. However, note that the average waiting time in peak hours is high and therefore this increase is relatively small.

## Discussion

In this work we present a novel method for routing calls in MHCCs. Our results show that our method, MCTS, can significantly improve the performance of MHCCs and consequently help meeting the increasing need for effective first aid emotional treatments. Specifically, our results show that our proposed method is significantly superior to the FCFS approach that is common in MHCCs today, and the difference is most notable in heavy call flow setting. We also observe that even a simple routing rule that relies on our predictions models, such as CMT, can improve the MHCC’s callers’ feedback in heavy call flow settings, yet to a more limited extent compared to MCTS. The bigger differences in the heavy call flow setting were somewhat expected, since that these settings require more routing decisions and include more options, and therefore there is great importance for the routing method. Recall that the heavy call flow setting is about 28% percent of the scenarios in ERAN, thus addressing it is crucial.

This is a first-of-its-kind work, addressing a challenge that was not tackled before: how to route calls in in MHCCs. Several routing methods have been proposed in the past to cope with similar challenges in the *general* contact centers. The methods can be roughly categorized into three main approaches:

1. Routing rules based on Queuing-theory [20], a mathematical discipline that studies the formation, function and congestion of queues. The research in the field is fundamentally based a queuing model, a real world application, such as a call center, and a mapping between the call theoretical model and the real world application. In the context of contact centers, researchers have used queuing theory in order to approximate fundamental aspects such as the arrival of requests and predicting the duration of handling a request. However, call centers cannot be mapped completely to queuing theory models, since their are several uncontrollable factors in call centers that cannot be captured by these models [13]. This approach assumes that callers can be divided immediately upon arrival into groups that are associated with unique characteristics, such as importance, service time and arrival rate [15, 34, 35]. With these assumptions, researchers developed theoretical queue models and analyzed the performance of routing rules by simulations under various constraints[15, 32, 34, 36]. For example, in [32], the authors present the following simple rule designed for environments where callers can be divided into groups and frequently abandon the service before being answered: Assuming each caller group *i* is associated with a holding cost per unit time, a service rate and the abandonment rate, which are denoted by $c_i$, $\mu _i$ and $\theta _i$, respectively. The callers with the highest score $c_i \mu _i / \theta _i$, is prioritized. They found that this simple rule is optimal for for achieving common objectives. In Subsection 2.3, we presented a solution that is inspired by this simple rule (CMT) and adjusted to the setting of MHCCs.
2. Machine learning-based routing. The underlying assumption in this approach is that the identification of caller type and the call characteristics is not trivial. Therefore the research focuses on the prediction of a caller’s service requirements and matches the caller to an appropriate agent who can provide that service. For example, Ilk et al. [17] address the problem of routing caller to agents in online chat-based contact centers. They propose a solution that utilizes problem description text, provided by the caller, in order to predict which agent type will best handle the caller’s problem. They found that their method improves customer routing accuracy and reduces service time and abandon rate in a live-chat contact centers of a S&P 500 firm.
3. Heuristic-based routing. Due to complexity of accurately modeling contact centers, in practice many contact centers, and specifically MHCCs, apply the First Come First Served (FCFS) heuristic, in which the longest-waiting caller is the next to be treated [37]. This simple method, by nature, aims to minimize the waiting time of customers. However, this method is often sub-optimal for achieving many of the contact-center’s objectives such as maximizing service quality [15, 37, 38]. The performance of various types of contact centers using FCFS and similar heuristic-based routing rules has been thoroughly analyzed in previous studies [22, 39].

Unfortunately, the mental healthcare domain is significantly different from general contact centers and therefore, the proposed methods from the first two approaches cannot be easily applied to it. Specifically, callers to MHCCs are commonly hard to categorize into actionable classes for several reasons; First of all, due to the nature of mental conditions, the caller to a MHCC often cannot accurately and briefly describe the exact issue or concern she is struggling with and, in many cases, the caller is dealing with multiple issues at once [8]. Furthermore, previous research has shown that, even when the issue of the conversation is explicitly indicated by the caller, the callers’ feedback on the conversation in the MHCCs depends, to a great extent, on the agent’s conversation skills, such as sensitivity to the callers sentiment and dealing with ambiguity, and is not necessarily related to the issue of the conversation [19]. This means that the classification of callers into the estimated mental concern or issue is in most cases not helpful for estimating important outcomes of the conversation such as the service time and expected service quality. In addition, MHCCs agents often receive equal training and therefore identifying each agent’s skill-sets is a complex task. These fundamental differences make the proposed solutions of call routing seem inadequate to most MHCCs.

The proposed routing method was superior to the other approaches, however the relatively high computational cost of MCTS may be a limitation, especially for large MHCCs having very often a heavy flow of callers. Nevertheless, the MCTS method is an *anytime algorithm*, meaning that it can return a valid routing decision even when limited by time and depth of search. When using this approach one must carefully consider the trade-off between the performance and the run time of the algorithms. Applying additional pruning techniques and using stronger computational power can significantly reduce the run time in practice.

An additional limitation of our approach is related to it’s acceptance by MHCCs and by the callers. An essential attribute of our solution is that it prioritizes calls over others, and this can seem unjustified to the users in many cases. In order to mitigate this concern, we intend to investigate various methods for explaining the routing decisions and explore additional methods that enforce fairness constraints. We further plan to extend our work and to investigate the performance of our approach in a larger MHCC and also in general, non mental health, contact centers.

In this study we used the callers’ explicit feedback in order to estimate the quality of the chats in ERAN. However, the callers’ feedback is a subjective measure and is viewed differently by various callers and in different environments. Furthermore, other MHCCs may not collect such feedback. This may be a limitation when applying the routing method in other environments. Nevertheless, There are many other possible indicators for estimating the chat’s quality: the overall impression of the agent from the caller at the end of the chat, the way the chat ended, etc. Further work is necessary in order to create a more general process for estimating of the chat’s quality in MHCCs and create new machine learning models for predicting the quality of the conversations. In addition, the objective of routing calls may vary between MHCCs, and thus applying our method to other MHCCs will probably require modifications.

Nevertheless, the novel general procedure that is proposed in this work is relevant and applicable in other MHCCs and can improve the performance of MHCCs in Israel and worldwide. Effective emotional first aid has the potential of preventing a deterioration in the mental state of the callers and specifically prevent suicide [8, 40]. Therefore, improving the performance of reduce the burden of emotional illness on the suffering individuals and in the economic and social burden in the population-level.

## Conclusions

MHCCs are a popular and effective way for aiding people who are struggling mentally. However, currently, many MHCCs operate in a simple first-come-first-serve scheduling policy and, consequently, they do not achieve optimal performance in their desired objectives . In this work, we propose a novel machine-learning based approach for routing callers to agents and show that this approach can bring about a significant improvement in both quality and quantity of the provided service . To that end, our novel approach utilizes novel prediction models, in order to estimate the long-term utility of different routing options. The evaluation of the study was performed by running realistic simulation, based on historical data. We plan to integrate the method into operational MHCC in future work. Our promising results show that in common scenarios (about 28%), replacing the common FCFS approach with our proposed routing method in MHCCs can significantly improve both the quality and the quantity of the served calls, which are their desired objectives. Consequently, applying our proposed method can reduce the burden of mental health issues on health systems.

## Figures and Tables

**Fig. 4:**
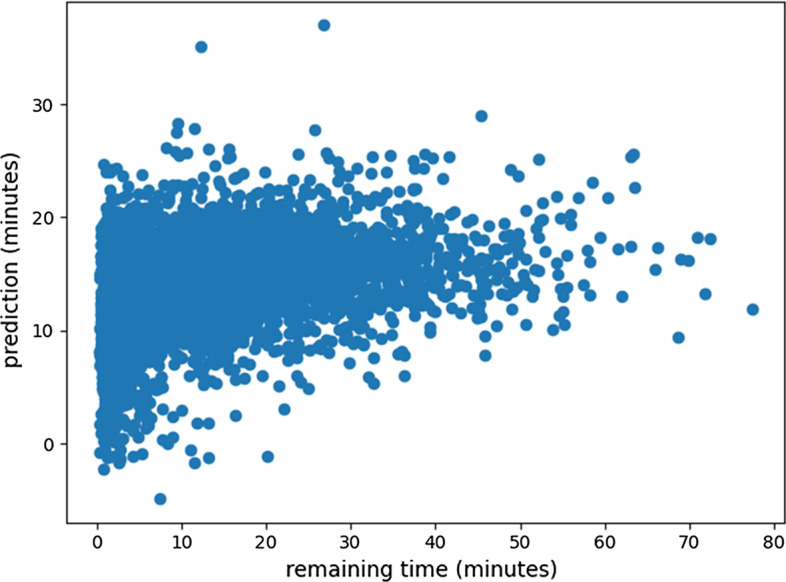
Remaining time regression prediction. The remaining time as a function of the real remaining time. We can observe that the prediction is relatively accurate when the chat is close to end

## Data Availability

The datasets generated and analysed during the current study are not publicly available due to privacy issues, but are available from the corresponding author on reasonable request. The code that was used for creating and evaluating the proposed routing methods is publicly available at the following link: *https://github.com/AkivaSinai/MHCC*.

## A Remaining time prediction

In Sect. 2.2.4 we described two prediction models for predicting the remaining time of a chat: A regression model and a binary classification model. Both models were necessary in our proposed scheduling solution. On the one hand, the regression model is more useful since it can improve prediction of the remaining duration at every given point in a chat, and the binary classification model is only usefull at predicting towards the end of the chat. On the other hand, the regression model fails to accurately predict conversation that are towards the end, which is crucial for improving the scheduling solution, as we will demonstrate below. Therefore, in our solution, we combine both model together in order to estimate the chat end time as follows: At a given decision point, the system initially activates the binary classification model. In case that the model predicts that the chat is close to end, the system updates the expected duration accordingly. Otherwise, the system activates the regression model and updates the remaining time of the chat accordingly.

### The regression model

In order to predict the expected remaining time we collected data describing uniformly generated random points from a conversation. for each conversation we sampled a single time-point. We then collected features describing the agent and the caller prior to the chat.

Similarly to above, we compared a few different regression models, and bench-marked the models’ performance against a naive predictor, that subtracted from the the duration of the chat until the sample’s time point form the agent’s average total duration.. Here we found that the Automatic Relevance Determination Baysien Regression (ARD Regressor) [41] outperformed all other models: We found that the RD Regressor scored 128.12 in the MSE measure while the baseline scored 371.08 ($\sim$ 65% improvement).

We later observed that our model was more accurate in predicting the remaining time as the conversation in the middle of the conversation than the prediction towards the end or at the beginning of the chat. Specifically, when the chat was 10–20 minutes from ending, the models average absolute error was 3.31 (mean squared error) and the standard deviation was 2.3, meaning that the prediction was relatively accurate. On the other hand, when more than 20 minutes were remaining, the prediction model average absolute error was 14.8 (s.d. = 9.67), and when less than 4 minutes were remaining the average absolute error was 9.61 (s.d.= 4.22). Therefore, for our simulation we divided the samples to different groups according to the actual remaining time and created distributions for generating error in each group separately. The relation between the actual remaining time and the prediction error are presented in Figure 4.

#### The binary classifier

we first set $k=3$, meaning that our model predicted if the chat will end in the next three minutes. Again we used features describing the conversations so far, and specifically features describing the most recent interactions in the conversation. Similar to the procedure described above, we tested a few different machine learning algorithms and used feature selection methods in order to improve the models’ performance. We found that the Random Forest algorithm outperformed all other ML algorithms and was able to predict balanced accuracy score of 0.89. We found that a few lexical features were highly influential in the prediction.

## B Patience estimation

Estimating patience is difficult and it cannot be accurately estimated with our data for a few reasons: First of all, our data does not include detailed description of the callers who abandoned. It only describes the general abandon rate per-hour and the average waiting time. In addition, callers willingness to wait in the queue is likely to be affected by the routing mechanism. Therefore, the patience of callers when the FCFS policy is applied, may not accurately reflect their patience when a different policy is applied.

Nevertheless, previous literature found that the actual waiting time of different types of callers is linearly correlated with their patience [22] and that the patience distribution can be modeled as an exponential distribution [32]. Therefore, in our evaluation, we estimated the patience of the callers by an exponential distribution and we adjusted the distribution’s rate parameter for each caller as follows: we first analyzed the actual waiting time in order to find which attributes have a significant effect on the waiting time. We found, for example, that the device the caller used during the connection has a significant influence on the waiting time. Specifically, callers who access the service with a mobile phone are significantly less patient (average waiting time= 266 seconds, s.d.= 523) than caller who used a desktop computer (average waiting time= 402 seconds, s.d.= 743.21). We also found that gender of the callers has a significant impact on the waiting time, female callers are in average more patient than the male callers(mean= 344.1 , s.d.= 665.9 vs. mean= 288.7 ,s.d.= 556 ), and in addition the age of the callers had an noticeable impact on the waiting time. For example, callers between ages 50 and 64 waited in average 520 seconds (s.d.= 506.45) while callers between ages 36 and 49 waited only 238 seconds (s.d.= 966.09). Therefore, in order to estimate the callers patience of callers is our case study, we divided the callers to groups associated with different distributions of patience: we divided according to the device, gender and age of the caller, a separate group for each possible combination. Then, we calculated the average waiting time across all callers in the group. Then, we set the distribution rate parameter to be the average waiting time. This distribution enabled us to generate an estimated waiting time for callers in our simulation.

## Abbreviations

MHCC: Mental health contact center.
FCFS: First come first served.
MCTS: Monte Carlo tree search.
WHO: World Health Organization.
ERAN: The Israeli mental health first aid association.

## Footnotes

1 https://en.eran.org.il/

2 e.g. https://www.riseuk.org.uk/get-help/first-steps/helplines-drop-ins, https://rappahannockareacsb.org/portfolio-view/suicide-prevention/
